# Adiabatic Heteronuclear Isotropic Mixing in Low-Field Nuclear Magnetic Resonance

**DOI:** 10.1021/acs.jpclett.5c03275

**Published:** 2025-12-26

**Authors:** Zefan Zhang, Christian Hilty

**Affiliations:** Chemistry Department, 14736 Texas A&M University, College Station, Texas 77843, United States

## Abstract

The heteronuclear isotropic mixing between ^1^H and ^19^F spins is demonstrated in low-field NMR. The efficient polarization transfer between nuclei expands the application range of low-field NMR in the chemical space, which is being made possible by new techniques of nuclear spin hyperpolarization. The isotropic mixing is demonstrated using a heteronuclear two-dimensional correlation spectrum of 3-fluoropyridine. An adiabatic WURST pulse achieved 50% transfer over the frequency difference of 2149 Hz in a magnetic field of 0.86 mT. While the efficiency of the DIPSI-2 was higher at 63%, it yielded a 26% less signal-to-noise ratio, compared to the WURST pulse experiment. In the presence of a 20% *B*_1_ miscalibration, the DIPSI-2 mixing efficiency degraded to 26%, whereas the adiabatic pulse performance was reduced by only 1% at an amplitude reduced by 62.5%. The smooth amplitude profile at low WURST order increases adiabaticity and mixing performance, when the pulse is sufficiently short to alleviate spin relaxation. The improved performance of the adiabatic pulse under these conditions is important for low-cost and ex-situ applications of low-field NMR spectroscopy. The observed polarization transfer efficiency in density matrix simulations of both sequences predicted the experimental values in both the optimized and miscalibrated conditions within 18%. The agreement indicates that the simulations can be used to design optimal mixing pulses for varied conditions in the low-field NMR experiments. Isotropic mixing sequences in this context may in the future be used for the characterization or identification of chemical compounds on the benchtop, in the field or in environmental applications.

## Graphical abstract

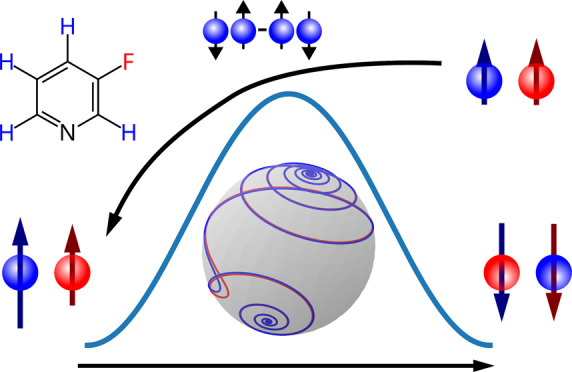

Despite the lack of resolution in chemical shift, low-field NMR in the milli-Tesla field range possesses unique advantages in several application scenarios, including Earth-field rheology in the environment,^1^ prospecting and molecular dynamic research using single-sided NMR,^2^ characterization of protein–ligand interactions,^3,4^ and low-field MRI in electromagnets.^5^ Even in the absence of chemical shift, a unique opportunity for chemical structure determination exists in the strong coupling regime, where *J*-couplings provide additional information.^6^ Despite the lack of chemical shift resolution, chemical compounds in a mixture can still be distinguished by two-dimensional (2D) spectroscopy if *J*-coupling patterns are resolved.^7^

We distinguish low-field NMR spectroscopy in a milli-Tesla magnetic field^8^ from that at zero field (micro-Tesla or lower)^9^ and typical benchtop spectroscopy (Tesla range).^10^ The distinction is important, as the low field by this definition resolves the Larmor frequencies of different nuclei, but not the chemical shifts of the same type of nucleus. In zero to micro-Tesla field, Larmor frequencies are insignificant and coherences evolve only due to *J*-couplings. Benchtop NMR most closely resembles the traditional high-field NMR by providing chemical shift resolution and treating each nucleus as a separate “channel” yielding its own spectrum.

Hyperpolarization makes NMR in the milli-Tesla regime readily possible. One crucial drawback of the low-field NMR is its low signal intensity. Hyperpolarization can increase the spin polarization to levels above thermal equilibrium and, in many cases, provide a nominal signal enhancement of a million-fold or more at a milli-Tesla magnetic field. Dynamic nuclear polarization and optical pumping have been applied to magnetic resonance imaging in a micro-Tesla field.^11,12^ Alternatively, the parahydrogen-based Signal Amplification by Reversible Exchange (SABRE) is well-suited to be implemented in a low-cost instrument, because it can be applied without requiring a high field at any point in the process.^13^ SABRE utilizes the singlet spin isomer of hydrogen, which can be easily and continuously enriched at low temperature. It transfers to the target molecule through binding to a polarization transfer catalyst simultaneously with a substrate molecule.^14^ The NMR signal enhancement by SABRE can be several hundred-fold compared to high-field NMR at 9.4 T, as high as 4100 times at 1 T,^15^ and proportionally larger at lower field. This method is applicable to a variety of nuclei such as ^1^H, ^19^F, ^13^C, or ^15^N.^16,17^ Couplings whose values fall within a wide range are manifested almost equally in a sequence of sufficiently long duration.^18^

Total correlation spectroscopy (TOCSY) is an application of isotropic mixing often used in high-field NMR determine correlations between spins and reveal single-and multibond connectivity.^19^ TOCSY in combination with benchtop NMR has further been described to identify molecules in chemistry^20^ and forensics applications.^21^ In zero field, mixing occurs during waiting periods.^22,23^ Shuttling of samples to zero field has also been used to achieve heteronuclear mixing for high-field NMR.^22,23^ Here, we demonstrate isotropic mixing for low-field NMR as a means to transfer magnetization throughout a spin system involving multiple types of nuclei and demonstrate its potential applications in organic molecules.

Several ways to achieve isotropic mixing in the presence of a frequency difference exist, including repetitive π pulses, phase-alternated composite spin-lock pulse trains, and adiabatic pulses.^24–26^ The isotropic mixing occurs when effective Hamiltonians of spins-to-mix are reduced to zero at the same time.^25^ The simplest form of mixing is continuous wave irradiation.^27^ Composite pulse trains, consisting of consecutive pulses with strictly defined duration and phase, were developed to achieve mixing at a lower pulse amplitude and wider frequency range. The WALTZ sequence is an early example, which presently is most commonly used for spin decoupling.^28^ DIPSI-2, a popular choice of an optimized composite pulse train, displays an even wider mixing profile.^25^ In contrast to composite pulse trains, adiabatic pulses such as WURST use a frequency sweep that may be combined with gradual amplitude changes to lock nuclear spins under quantum mechanical adiabatic conditions.^26^ Adiabatic pulses are resilient toward frequency and *B*_*1*_ miscalibration or drift.^26,29^ These pulses are normally applied at high field for homonuclear mixing. However, the difference in Larmor frequency in low-field NMR is small enough that an adiabatic pulse can cover multiple nuclei. Adiabatic pulses have already been demonstrated for *B*_0_ and *B*_1_ inhomogeneity-tolerant excitation and refocusing in single-sided low-field NMR.^30,31^

In the following, we demonstrate adiabatic isotropic mixing of ^19^F and ^1^H spins. TOCSY spectra with SABRE hyperpolarization and simulations are used to quantify the mixing performance and demonstrate a superior tolerance of the adiabatic pulses to external field inhomogeneity.

The WURST-TOCSY experiments (Figure 1a) were performed by applying a WURST adiabatic pulse in the mixing stage of a TOCSY pulse sequence. To ensure the proper mixing of heteronuclear spins, full adiabatic passages are required for all spins involved.^26^ The frequency sweep range of the WURST pulse (Figure 1b) covered both ^1^H and ^19^F Larmor frequencies with sufficient margins to ensure proper heteronuclear mixing, and is expected to achieve the mixing with one pulse. The signal strengths of ^1^H and ^19^F are different due to differences in SABRE efficiency and possibly the tuned circuit of the receiver coil.

**Figure 1:**
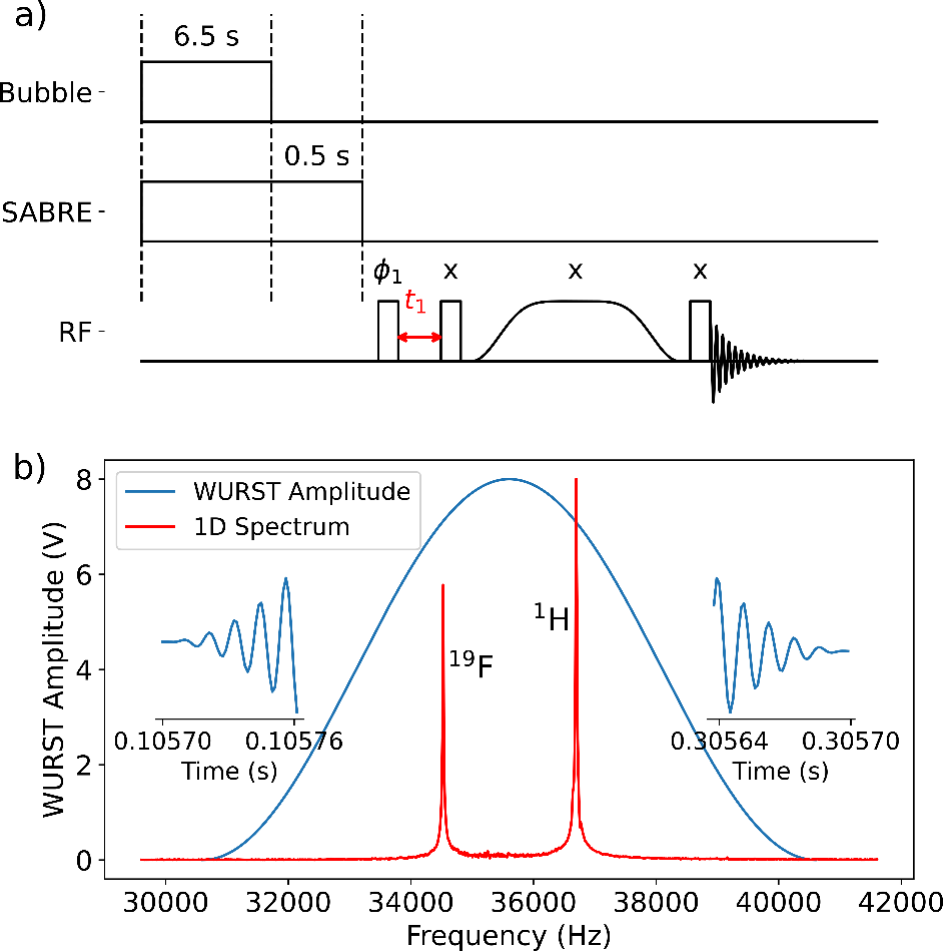
(a) WURST-TOCSY pulse sequence. The first two lines indicate bubbling of parahydrogen into the sample and application of the SABRE polarization field, respectively. The rectangles on the RF channel represent the tailored pulses, whose effect is a net 90° rotation for both ^1^H and ^19^F. The shaped pulse is the WURST pulse, which covers both the ^1^H and ^19^F frequencies. Phases are indicated above the pulses. The pulse phase ϕ_1_ = *x* -*x*. ϕ_1_ and the receiver phase are additionally incremented for each acquisition according to the States-TPPI protocol. b) Amplitude-frequency curve of the second order WURST pulse (blue) overlaid on a 1D spectrum of 3-fluoropyridine (red) to show the center frequency and sweep range. The insets to the left and right show the start and end of the time-domain function of the WURST pulse, respectively.

Heteronuclear total correlation spectra were measured from hyperpolarized ^19^F and ^1^H spins of 3-fluoropyridine using the WURST-TOCSY pulse sequence. Two of the time domain signal traces are shown in Figure 2. They include the pulses and the signals induced by nuclear spins during different time periods.

**Figure 2:**
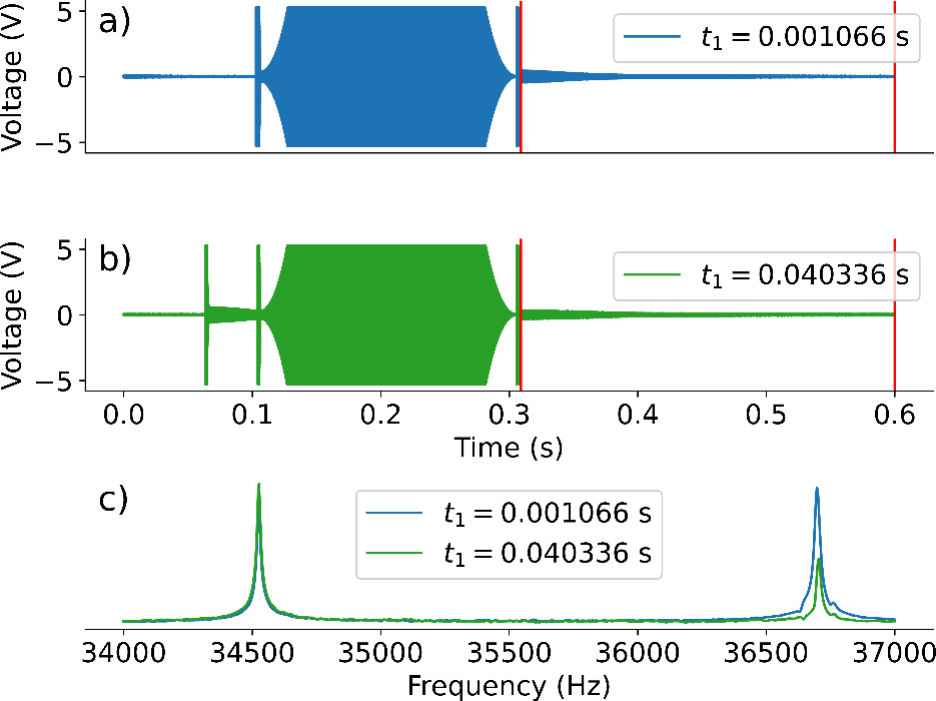
Acquired time-domain signal of the (a) 3rd and (b) 175th scan of a WURST-TOCSY experiment, with an adiabatic pulse amplitude of 640 Hz and order of 2. Both signals are from the first step in the phase cycle. (c) Fourier-transformed spectra of the two signals. The pulse amplitudes, which appear before the Fourier-transformed range, to the left of the first vertical red line, are clipped because of the receiver dynamic range. The first two pulses in panel (a) are too close to visually differentiate.

The traces shown are from the same step of the phase cycle to demonstrate a successful mixing through the modulation of signals by scalar couplings at differing *t*_1_ evolution times. The part of the signal following the end of the ring-down of the last pulse to the end of the experiment was Fourier transformed, as indicated by the vertical red lines in the figure. In the resulting spectra, the ^19^F and ^1^H peaks are observed at 34 536 and 36 701 Hz, respectively. The modulation of the intensities of the two peaks by the incremented *t*_1_ time, shown in Figure 2c, is indicative of the presence of the ^19^F–^1^H heteronuclear scalar coupling.

In the *B*_0_ magnetic field of 0.86 mT, the Larmor frequencies of the two nuclei are sufficiently close to enable simultaneous excitation and mixing using a single pulse, instead of requiring a second RF channel. The mixing pulse needs to possess a sufficient minimum adiabaticity, which will provide spin-locks throughout the passage. The WURST adiabatic pulses, which were originally developed for high-field application, provide a high adiabaticity in a narrow band in the center of the sweep range.^32^ Thus, the sweep range of the WURST pulse in the low-field pulse sequence was set to 10 kHz, while the ^1^H and ^19^F signals were off-resonance from the center by approximately 1.1 kHz. In comparison, a faster sweep of 2 kHz in 32 ms was reported to achieve a good inversion efficiency of −0.999 in test experiments at Earth field.^30^

The 2D spectrum (Figure 3) shows two crosspeaks and two diagonal peaks. The spectra exhibit a signal-to-noise ratio on the order of 3000 (instrument noise region in Figure 3). Noise bands in the indirect dimension, also known as *t*_1_ noise, appear vertically near the two peaks. This noise is primarily due to instabilities in signal intensities, with peaks exhibiting a signal-to-noise ratio (SNR) on the order of 100 with respect to this noise (Figure 4, black trace).

**Figure 3:**
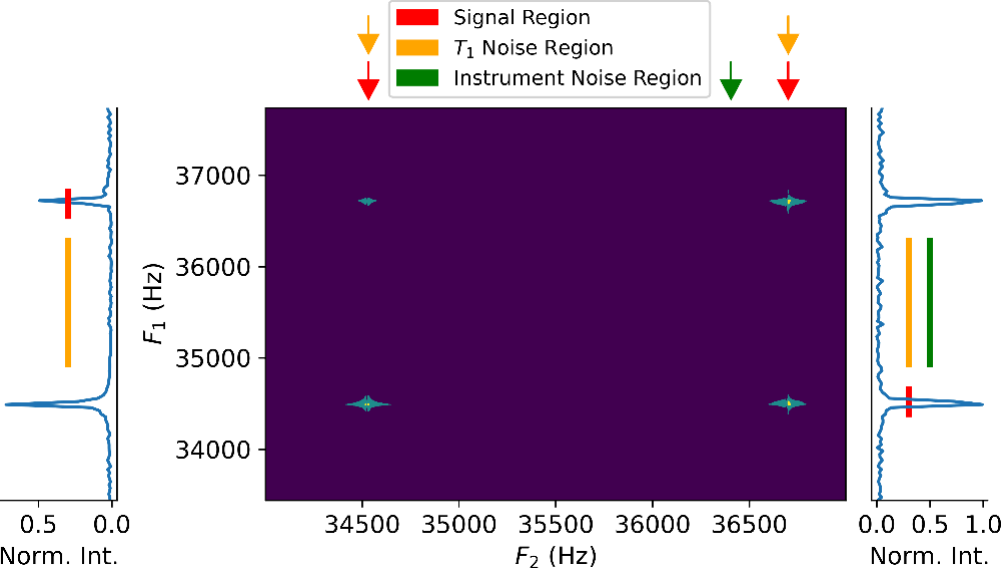
2D WURST-TOCSY spectrum with a WURST pulse duration of 200 ms, order of 2 and amplitude of 640 Hz. This result constitutes the 1^st^ entry in Table S1. The slices for sampling signal, *t*_1_ noise, and instrumental noise are indicated by the arrows at the top and bars at the sides. Vertical slices of the spectra taken on the direct axis at the frequencies of ^19^F (left) and ^1^H (right) are shown. The 2D spectrum is shown with logarithmic contour levels, and the slices are on a linear scale.

**Figure 4:**
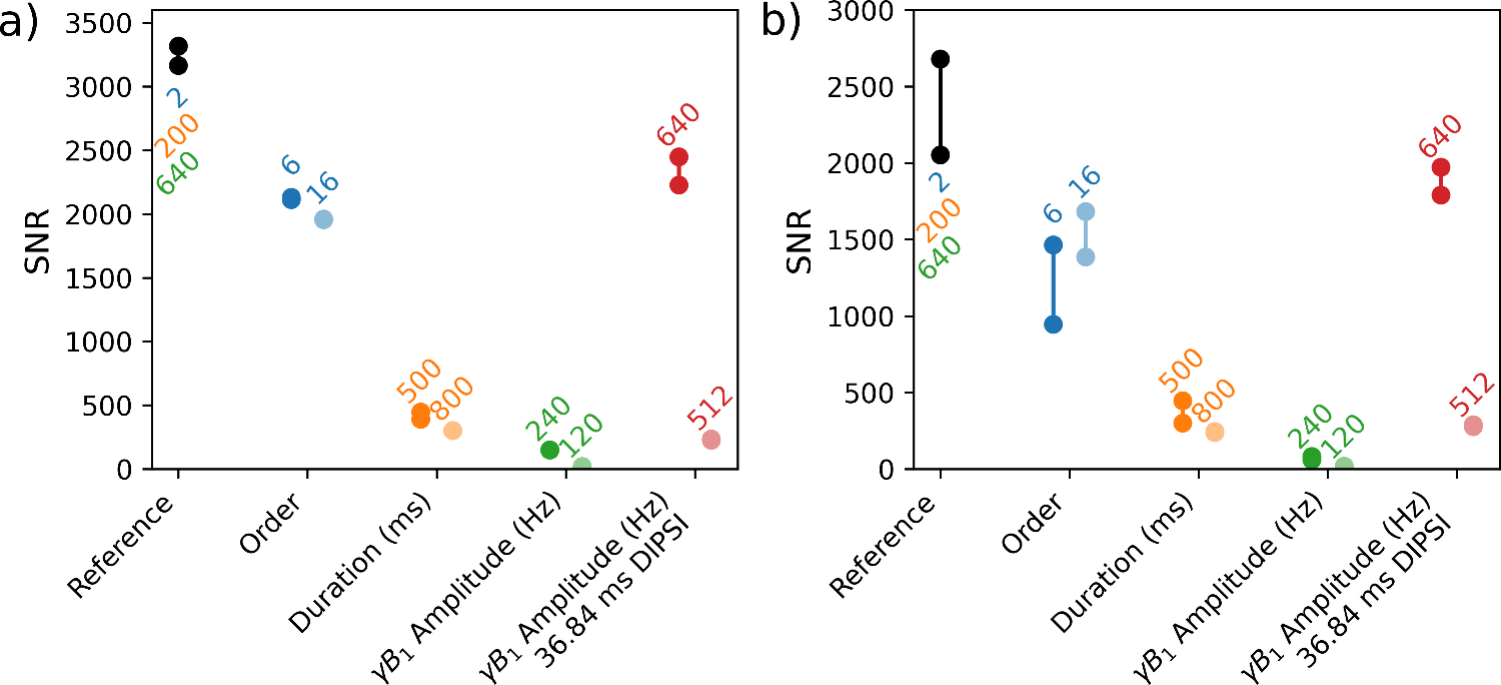
Cross-peak instrumental signal-to-noise ratios of the experiments by different WURST and DIPSI-2 pulse settings of (a) ^1^H and (b) ^19^F peaks. The 200 ms duration, 640 Hz amplitude and *N* = 2 WURST pulse is used as reference, colored black. Three control groups of pulse order, duration in milliseconds and *γB*_1_ amplitude in Hz are compared to the reference with the variable parameter labeled and colored. Optimal and *B*_1_ miscalibrated DIPSI-2 are compared to the WURST experiments, with the amplitude of *γB*_1_ (in Hz) labeled. The two connected data points are two repeats, listed in Tables S1 and S2.

The adiabatic pulse in Figure 1 was chosen with the lowest possible order of 2. A calculation of the adiabaticity during the pulse (eqs S1–S6) suggested that the smoothly increasing amplitude improves the adiabatic behavior near the beginning and end of the pulse. Therefore, a much narrower relative sweep range can be used compared to typical applications of adiabatic pulses in high-field NMR, alleviating the spin relaxation during the pulse. These properties of the pulse were corroborated by additional experiments (Figure 4). Among pulse orders of 2, 6, and 16 (Figure 4, black vs blue), both ^1^H and ^19^F peaks indeed show the best instrumental SNR at the lowest pulse order of 2. Both ^1^H and ^19^F cross further peaks exhibited decreasing instrumental SNR at increasing mixing pulse duration (Figure 4, black vs orange). With the increase in duration, the slower frequency sweep increases adiabaticity and, in theory contributes to a more efficient mixing. However, the signal loss caused by relaxation outweighs the improvements on adiabaticity.

Reducing the RF power of the WURST pulses decreases the ability to hold spin-lock, reflected in a decreasing adiabaticity and performance. In the data shown, the pulse amplitude was decreased so γ*B*_1_ changed from 640 Hz to 240 and 120 Hz (Figure 4, black vs green), which corresponds to a calculated minimum adiabaticity of 39.79, 5.72 and 1.43, respectively. The spectrum with the 240 Hz pulse (Figure S2a) shows a much lower instrumental SNR (Figure 4, green) compared to the reference 640 Hz spectrum (Figure 3), with only ^1^/_22_ of ^19^F and ^1^/_32_ of ^1^H instrumental SNR. The spectrum with the weakest, 120 Hz pulse (Figure S2b) is observed to have very weak cross and diagonal peaks. Altogether, the comparisons indicated that the lowest order pulse with shortest duration and highest pulse amplitude led to the best signal.

A parallel experiment to test the RF offset resistance of the pulse program was carried out with a −100 Hz frequency miscalibration of the adiabatic pulse, and was found to have minimal effect on the spectrum (Figure S3). No observable mirror peak from quadrature artifacts caused by imperfect phase cycling suppression is seen on the asymmetrical pattern on the indirect axis. The instrumental SNR of the frequency miscalibrated spectrum were measured to be 2712.51 and 1491.60 for ^19^F and ^1^H, only slightly lower than the control experiment shown in Figure 3.

The adiabatic mixing was further compared to DIPSI-2, an established nonadiabatic mixing sequence that employs a series of hard pulses.^25,33^ The DIPSI-2 sequence was used in place of the WURST pulse to compare their performances. The DIPSI-2 TOCSY experiment (Figure S4) was observed to have an instrumental SNR (Figure 4, black vs red), which was, however, 26% lower than the optimal 200 ms, 640 Hz, *N* = 2 WURST experiment. The isotropic mixing efficiency offset-dependent profile can be used to quantify the mixing performance.^26,29,34^ The mixing efficiencies are calculated as the proportion of transferred magnetization in the overall magnetization of the final spin system state (*P*_cross_/(*P*_cross_ + *P*_diagonal_)). In the formula, *P* is the SNR of the cross or diagonal peaks of the same nuclei on the direct axis. The efficiencies are found at around 0.5 and 0.3 for ^1^H and ^19^F (Figure S5), respectively, independent of the mixing time in the range tested. The 200 ms is already sufficient for mixing, considering that the strongest coupling in 3-fluoropyridine is 8.79 Hz.^35^

Isotropic mixing efficiency simulations based on density matrix evolution were used to compare to the experiments. The adiabatic full passage inversion and isotropic mixing are demonstrated in Figures S6 and S7. The isotropic mixing efficiency frequency-offset profiles are plotted in Figure 5. The zero-offset efficiencies of DIPSI-2 and WURST were simulated and found to be 0.98 and 0.84 at the experiment *B*_0_ strength with the same *B*_1_ as the experiments, and the efficiencies at the offset of ^1^H and ^19^F spins to the spectral center were 0.81 and 0.57 (Figures 5a and 5b). The experimental heteronuclear mixing efficiencies of DIPSI-2 and WURST are calculated to be 0.63 and 0.50 (Figure S5). The slightly lower experimental efficiency could be attributed to the additional weaker ^1^H–^19^F coupling constants, which are absent in the two-spin simulated model.

**Figure 5:**
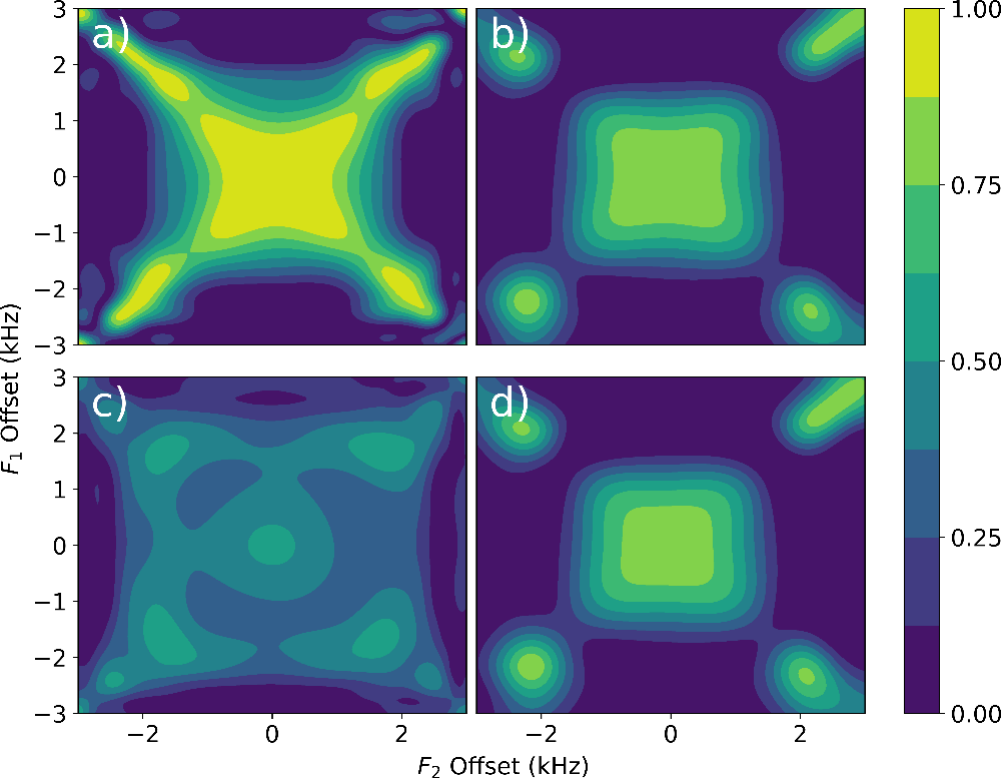
Simulated offset-dependent isotropic mixing efficiency profile of (a) DIPSI-2, (b) WURST pulse, (c) −20% *B*_1_ miscalibrated DIPSI-2, and (d) −20% *B*_1_ WURST pulse. The pulses were simulated on a two-spin system where *J* = 10 Hz. The reference DIPSI-2 pulse used a *γ* *B*_1_ of 640 Hz, and the reference WURST used a pulse duration of 200 ms, order of 2 and a maximum *γ* *B*_1_ of 640 Hz.

Although the efficiency of DIPSI-2 is higher, the adiabatic isotropic mixing pulse demonstrates advantages in imperfect *B*_1_ fields. When *B*_1_ suffers a −20% *B*_1_ miscalibration, the zero-offset efficiencies of DIPSI-2 and WURST were simulated at 0.52 and 0.81, and the efficiencies at the offset of ^1^H and ^19^F spins to the spectral center were 0.27 and 0.53 (Figures 5c and 5d). The experimental mixing efficiencies of DIPSI-2 were also found to agree with the simulations, degrading from 0.63 to 0.26 with *B*_1_ miscalibration. In contrast, the experimental efficiency of WURST at −62.5% *B*_1_ miscalibration is reduced from 0.50 to 0.49, only 2% less. The *B*_1_ miscalibration tolerance of the WURST pulse allows applications such as single-sided NMR and bulky or time-sensitive NMR experiments where pulse calibration is not possible.

The potential application of the WURST pulse for isotropic mixing of ^1^H with X = ^13^C or ^15^N spins in organic and biological molecules is investigated in the following. These molecules may contain dilute X spins, such as ^13^C at 1% natural abundance. The heteronuclear mixing allows the polarization to transfer through both ^1^H–^1^H and ^1^H–X networks. Therefore, organic molecules with a ^1^H-bonded carbon network can still manifest long-range mixing in the absence of direct ^13^C connectivity. A lower receptivity of the heteronucleus reduces the SNR. Compared to the described experiments, where a ^1^H SNR of >3000 was observed, ^13^C signals would be expected to be observable with an SNR > 7, considering a 400 times lower receptivity.

The frequency difference between ^1^H and X would be larger than in the case of ^19^F. Simulated mixing efficiency profiles of WURST and DIPSI-2 covering a corresponding wider range of offset relative to the frequency are illustrated in Figures S8–S10. The field strengths were chosen as the 0.86 mT of the present experiments, 63 μT corresponding to Earth field, and 8.6 μT representing a shielded ultralow field instrument. The mixing efficiency of the WURST pulse was found to increase at lower *B*_0_ field strength and to decrease at higher frequency offset. To mix ^1^H and X, the required wider WURST sweep range increases the sweep rate if the time is kept constant below the relaxation time. Increasing the *B*_1_ amplitude is then necessary to meet the adiabatic condition.

Among the simulated conditions, the WURST pulse achieved the best mixing efficiencies of 0.67, 0.52, and 0.35 for ^1^H–^31^P, ^1^H–^13^C, and ^1^H–^15^N, respectively, at 8.6 μT. With the same pulse amplitude, DIPSI-2 achieved less than 5% of those values at optimal settings. The difference in mixing efficiency between the chosen coupling constant of *J* = 10 Hz, which would correspond to a multibond ^1^H–X coupling and *J* = 140 Hz for a single-bond ^1^H–^13^C coupling was negligible under a pulse duration of 200 ms (Figure S11). The lower efficiency of DIPSI-2 is explained by the large difference of the gyromagnetic ratio of the involved nuclei, requiring a broader bandwidth to satisfy the Hartmann–Hahn condition.

The simulations illustrate that an optimal set of parameters can be found for a range of use cases. In higher fields, *B*_1_ amplitudes exceeding experimental constraints may be required. For example, the *B*_1_ that was used in the experiments (Figure 1) would not be sufficient for mixing ^1^H and ^13^C. This application would become possible if the amplitude were increased by 4.5 times. Second, the simulations indicate that mixing of spins with a large difference in gyromagnetic ratio, such as ^1^H and ^13^C, becomes easier at lower field.

Optimizations of the mixing profiles can be envisaged. The adiabatic mixing efficiency profile can be normalized into an ideal square shape if multiple adiabatic pulses are composed of a phase scheme and expanded by a super cycle, for instance, P5, P9 and P5M4,^29^ despite the fact that a single adiabatic pulse can still achieve isotropic mixing in a narrow offset range.^36^ However, in the context of low-field NMR, such super cycles require a longer time to execute than the 800 ms WURST experiment and will be subjected to a greater relaxation loss, in the present experiment, making a single adiabatic passage a better choice.

In summary, the experiments demonstrate that sufficiently powered adiabatic pulses could achieve heteronuclear mixing in the mT magnetic field range. A WURST pulse with lowest order of 2 retained adiabaticity at a narrow sweep range due to its smooth *B*_1_ amplitude profile. A short mixing pulse duration decreases the relaxation loss and improves the mixing performance. The mixing efficiency was measured and referenced to that of the nonadiabatic DIPSI-2 sequence. The mixing efficiency of WURST was found to be insensitive to pulse miscalibration both experimentally and in density matrix simulations. Adiabatic pulses with smooth amplitude profiles and short duration appear ideal for efficient isotropic mixing for instruments with *B*_0_ and *B*_1_ magnetic field inhomogeneity, seen in many applications of low-field NMR. It may be applied to distribute spin magnetization in H–X spin systems, potentially increasing the applicability of low-field NMR with common organic molecules.

## Supplementary Material



## Notes

Experimental methods, signal-to-noise ratio of TOCSY spectra, calculation of time-dependent adiabaticity of WURST pulse, test experiments, isotropic mixing efficiency measurements, isotropic mixing efficiency simulation, wide range isotropic mixing efficiency simulation (PDF)

## ABBREVIATIONS

NMR: nuclear magnetic resonance
SABRE: Signal Amplification By Reversible Exchange
TOCSY: total correlation spectroscopy
