# Supplementary material for: Adiabatic Heteronuclear Isotropic Mixing in Low-Field Nuclear Magnetic Resonance
Source: J Phys Chem Lett. 2025 Dec 26;17(1):48–53. doi: 10.1021/acs.jpclett.5c03275 (PMC12794145; doi:10.1021/acs.jpclett.5c03275)
Supplement: Supplementary file 1 [file jz5c03275_si_001.pdf]

Supporting Information for

Adiabatic Heteronuclear Isotropic Mixing in Low-field NMR

Zefan Zhang and Christian Hilty*

Chemistry Department, Texas A&M University, College Station, TX 77843, USA

e-mail: chilty@tamu.edu

Table of Contents

1. Experimental Methods....................S2
   1.1 Sample Preparation....................S2
   1.2 SABRE Low-Field NMR Spectroscopy....................S2
2. Signal-to-Noise Ratio of TOCSY Spectra....................S3
3. Calculation of Time Dependent Adiabaticity of WURST Pulse....................S5
4. Test Experiments....................S7
5. Isotropic Mixing Efficiency Measurements....................S8
6. Isotropic Mixing Efficiency Simulation....................S8
7. Wide Range Isotropic Mixing Efficiency Simulation....................S10
8. References....................S13

1. Experimental Methods

1.1 Sample Preparation

The sample for SABRE NMR contained 0.5 mM of chloro(1,5-cyclooctadiene)[4,5-dimethyl-1,3-bis(2,4,6-trimethylphenyl)imidazol-2-ylidene] iridium(I) (Strem Chemicals Inc., Newburyport, MA), 5 mM of 3-fluoropyridine (Ambeed Inc., Arlington Hts, IL) and 5 mM of d_6-dimethyl sulfoxide (99.9% deuterated, Cambridge Isotope Laboratories, Inc., Andover, MA) in methanol (VWR International, Radnor, PA). The solution was a transparent light-orange color. Hydrogen gas of grade 5.0 (Linde US, Danbury, CT) was converted into parahydrogen at 29 K temperature and 8.3 bar pressure in a cryocooler (Advanced Research Systems, Macungie, PA).

1.2 SABRE Low-Field NMR Spectroscopy

The NMR experiments were performed in a custom-made low-field NMR spectrometer as previously described.[1,2] A tetra-coil supplied a constant B_0 field of 0.86 mT. The RF irradiation signal and NMR signal were generated and received by a PCIe-6363 data acquisition board (NI, Austin, TX) at a rate of 800,000 samples per second. The radio frequency (RF) receiver coil was tuned to the Larmor frequency of ^{19}F spins, and the excitation coil was not tuned. The quality factor of the RF receiver coil is measured to be 21.32. For the SABRE-NMR experiment, a 1.5 mL aliquot of the liquid sample was loaded into a 10 mm NMR tube, connected to the parahydrogen delivery system and pressurized. Parahydrogen gas was then bubbled into the sample for 5 minutes at a flow rate of 0.1 standard liter per minute (SLPM). The sample was observed to become a colorless transparent liquid during this time.

The WURST-TOCSY experiments started with bubbling parahydrogen gas for 6.5 s at a flow rate of 0.1 SLPM. A constant magnetic field of 6.4 mT was activated for 7 s, starting simultaneously with the bubbling, to facilitate the SABRE process. The RF pulse sequence was timed for the second pulse to occur 0.7 s after turning off the 6.4 mT magnetic field, such that NMR signals appeared at the same time point in each scan. The first pulse occurred at a variable time dependent on the t_1 evolution time and additionally was phase shifted to follow the States-TPPI protocol. The experiment utilized a WURST pulse of order 2, 6 or 16 in the place of the conventional isotropic mixing sequence.[3,4] The applied amplitude of the adiabatic pulse was 3.00, 1.12 or 0.56 V, which were expressed as 640, 240 and 120 Hz as the γB_1 value of an on-resonance pulse on ^{1}H. The frequency sweep was from -5 to +5 kHz with respect to the center frequency of 35.6 kHz. DIPSI-2 reference experiments were performed with dual on-resonance hard pulses, with individual amplitudes of 3.00 V, same as the strongest WURST amplitude, which is expressed as 640 Hz. The DIPSI mixing time was 36.84 ms.[5] A second set of experiments was performed with a miscalibrated DIPSI-2 pulse amplitude, which was set to 2.40 V and expressed as 512 Hz. All other pulses were the same as in the DIPSI-2 reference experiment. The incremented t_1 evolution time started from 0.142 ms, with a step size of 0.462 ms for 128 steps.

All excitation pulses in the pulse sequence were "tailored", consisting of a single sine wave with square envelope, with a frequency between the ^{19}F and ^{1}H Larmor frequencies, to circumvent the problem of simultaneous hard excitation on ^{1}H and ^{19}F spins when the length of a hard pulse covering the required frequency difference would be shorter than a period of the radio-frequency.

The tailored pulse functioned by setting the radio-frequency B_1 field values identical to the residual magnetic field ΔB_0 in the rotating frame to achieve effective fields at 45 degree angle from z axis. The effective field then caused a 90° rotation in a time $\sqrt{2}t$, where t is the pulse length of an on-resonance 90° pulse of the same amplitude. The tailored pulses induced opposite phases of ^{1}H and ^{19}F in-phase coherences, compatible with the TOCSY pulse sequence. The frequency difference to each nucleus was proportional to the corresponding gyromagnetic ratio, which is 40.078 and 42.577 MHz·T^{-1}, respectively. The applied amplitude of the tailored excitation pulse was calibrated at 5.2 V, so the B_1 values were identical to the rotating frame ΔB_0 values for both ^{1}H and ^{19}F, at 1107 and 1042 Hz respectively.

The NMR datasets were processed using an in-house developed Python program (Python Software Foundation, www.python.org).[6] Excerpts starting from 0.01 s after the last pulse to the end of the acquisition, a total of 0.29 s, were used as time-domain data and analyzed. A real-valued Fourier transform was performed on the directly acquired axis. Subsequently, the real values of the 1D spectra were retained for the indirect axis and processed to achieve frequency discrimination following the States-TPPI protocol.

2. Signal-to-Noise Ratio of TOCSY Spectra

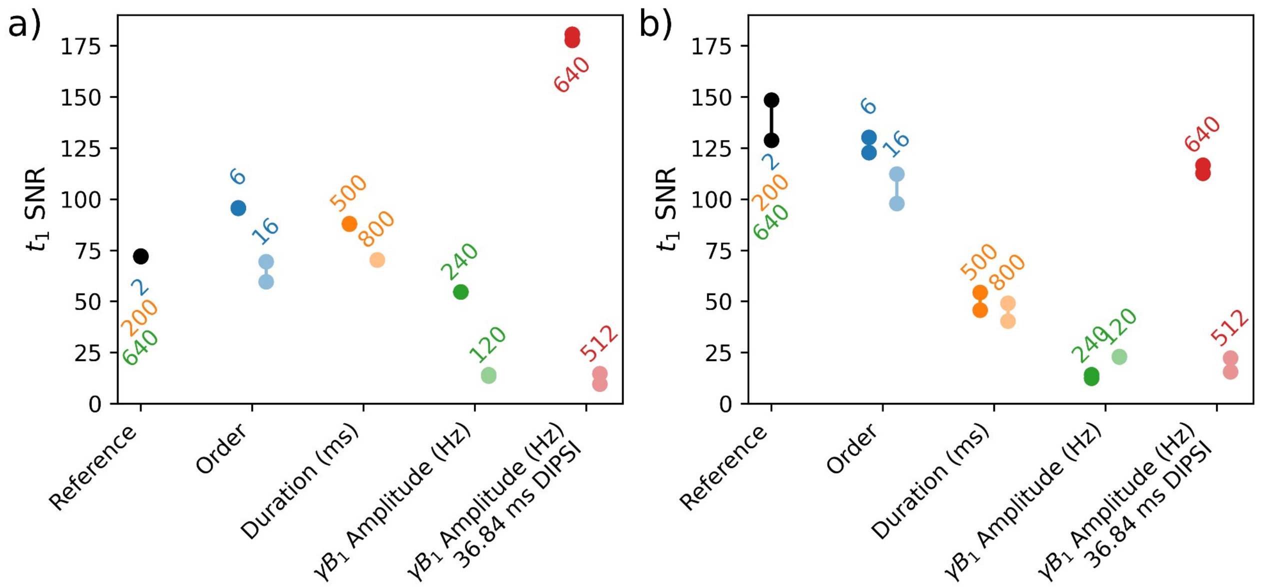

Figure S1: Cross-peak t_1 *signal-to-noise ratios of the experiments by different WURST and DIPSI-2 pulse settings of a)* ^{1}*H and b)* ^{19}*F peaks. The 200 ms duration, 640 Hz amplitude and* N = *2 WURST pulse is used as reference, colored black. Three control groups of pulse order, duration in milliseconds and* γB_1 *amplitude in Hz are compared to the reference with the variable parameter labeled. Optimal and* B_1 *miscalibrated DIPSI-2 sequences are compared to the WURST experiments, with* γB_1 *amplitude in Hz labeled and colored. The two connected data points are two repeats, listed in Table S1 and S2.*

The signal-to-noise ratio (SNR) of WURST TOCSY experiments are calculated and compared to DIPSI-2 TOCSY experiments. The signal of ^{1}H and ^{19}F spins are calculated separately as the

maximum value around the cross peak positions, shown in Figure 3 in red. The t_1 noise of ^{1}H and ^{19}F spins is calculated by taking the standard deviation of spectral data on a central segment between cross and diagonal peaks, shown in Figure 3 in orange. The instrumental noise from the electronic noise of the RF receiver coil, preamplifier and the data acquisition device are calculated as a standard deviation of spectral data on a segment away from the peaks, shown in Figure 3 in green.

Table S1: Cross peak signal-to-noise ratios (t_1 *and instrumental noise) of the experiments with different WURST and DIPSI-2 pulse parameters, repeat 1. The time indicates the duration of the pulse, the frequency denotes the* γB_1 *value of an on-resonance pulse on* ^{1}*H of the same amplitude.* N *denotes the order of the WURST pulse. The 36.84 ms duration DIPSI-2 pulse uses the amplitude of 640 Hz, as the strongest WURST-2 amplitude, and the miscalibrated DIPSI-2 ("miscal") uses 20% weaker amplitude of 512 Hz.*

	WURST							DIPSI-2	
	200 ms, 640 Hz, N=2	500 ms, 640 Hz, N=2	800 ms, 640 Hz, N=2	200 ms, 640 Hz, N=6	200 ms, 640 Hz, N=16	200 ms, 240 Hz, N=2	200 ms, 120 Hz, N=2	36.84 ms	36.84 ms miscal
^{1}H, t_1	71.91	88.03	70.19	95.51	69.50	54.63	14.17	180.7	14.69
^{1}H, instrumental	3317	391.5	301.5	2113	1957	150.6	20.31	2449	226.2
^{19}F, t_1	148.4	54.32	49.17	122.7	112.2	14.17	22.82	116.6	15.54
^{19}F, instrumental	2679	447.2	238.6	1465	1682	82.45	18.52	1974	289.5

Table S2: Cross peak signal-to-noise ratios (t_1 *and instrumental noise) of the experiments with different WURST and DIPSI-2 pulse parameters, repeat 1. The time indicates the duration of the pulse, the frequency denotes the* γB_1 *value of an on-resonance pulse on* ^{1}*H of the same amplitude.* N *denotes the order of the WURST pulse. The 36.84 ms duration DIPSI-2 pulse uses the amplitude of 640 Hz, as the strongest WURST-2 amplitude, and the miscalibrated DIPSI-2 ("miscal") uses 20% weaker amplitude of 512 Hz.*

	WURST							DIPSI-2	
	200 ms, 640 Hz, N=2	500 ms, 640 Hz, N=2	800 ms, 640 Hz, N=2	200 ms, 640 Hz, N=6	200 ms, 640 Hz, N=16	200 ms, 240 Hz, N=2	200 ms, 120 Hz, N=2	36.84 ms	36.84 ms miscal
^{1}H, t_1	72.19	87.84	70.32	95.74	59.56	54.58	13.50	177.6	9.62
^{1}H, instrumental	3166	445.8	302.1	2131	1961	150.9	20.19	2226	235.4
^{19}F, t_1	128.8	45.79	40.41	130.2	97.85	12.38	23.03	112.7	22.33
^{19}F, instrumental	2054	299.9	245.3	946.4	1387	61.83	17.51	1790	278.1

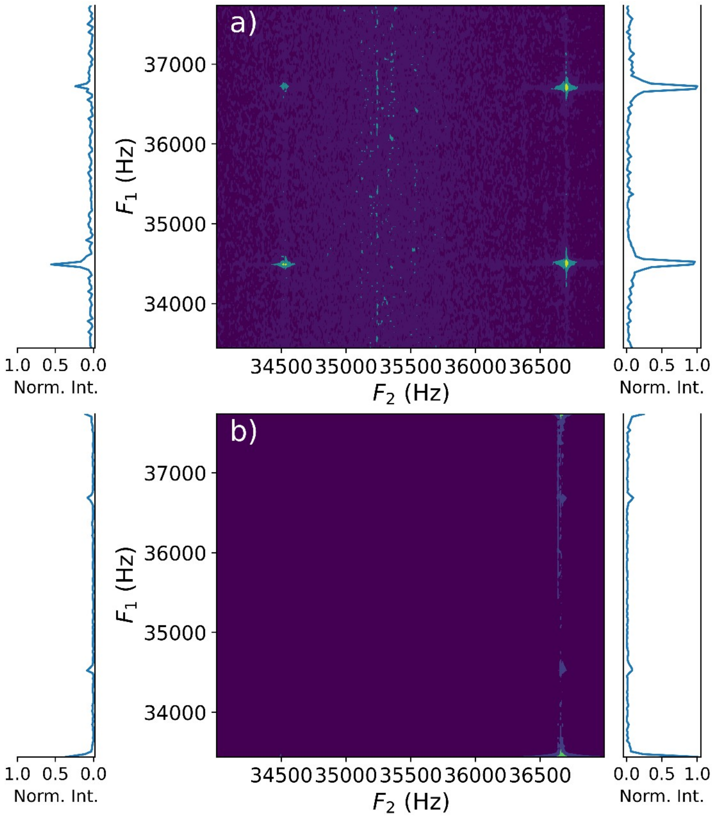

Figure S2: WURST-TOCSY spectra demonstrating the effects of adiabaticity with a pulse amplitude of a) 240 Hz and b) 120 Hz. The with pulse order was 2 and pulse duration was 200 ms. The minimum adiabaticity were calculated as 5.72 and 1.43 respectively. Vertical slices of the spectra taken on the direct axis at the frequencies of ^{19}F (left) and ^{1}H (right) are shown. The contours are shown on a logarithmic scale and the slices are on a linear scale, all normalized to the strongest shown signal.

3. Calculation of Time Dependent Adiabaticity of WURST Pulse

The adiabaticity, Q,[7,8] was calculated in a time-dependent manner for the entire duration of the pulse. The calculations were performed for pulses covering the ^{19}F and ^{1}H Larmor frequencies as described in the text. The below equations and procedures were used.

The WURST pulse is a chirp pulse whose frequency is swept linearly by time from the starting frequency to end frequency and modulated by a profile function *I(t)*.[3]

$$I(t)=I_{max}A(t)\sin(2\pi ft+\varphi(t)) \tag{S1}$$

$$A(t)=1-\left|\cos\left(\frac{\pi t}{\tau_w}\right)\right|^N \tag{S2}$$

The *A(t)* represents the amplitude function. The I_{max} is the maximum amplitude, the *t* is the time, the τ_w is the pulse duration, the *N* is the order of the WURST pulse and the *f* is the center frequency. The *φ(t)* is a quadratic phase function that transposes the sine function into a chirp function whose frequency varies linearly by time.

$$\varphi(t)=\pm 2\pi\left(\frac{\Delta}{2}t-\frac{\Delta}{2\tau_w}t^2\right) \tag{S3}$$

The Δ represents the sweep frequency range. The sign of the phase function determines the sweep direction, and a negative sign, indicative of low to high frequency, was used in the experiment.

The adiabaticity is calculated as the ratio of the effective field to the derivative of effective field angle to time, in the rotating reference frame.

$$Q(t)=\frac{|\gamma B_{eff}|}{\left|\frac{d\phi}{dt}\right|}=\frac{\gamma\sqrt{A^2(t)+\left(B_0-\frac{\omega(t)}{\gamma}\right)^2}}{\frac{d}{dt}\left(\arctan\left(\frac{A(t)}{B_0-\frac{\omega(t)}{\gamma}}\right)\right)} \tag{S4}$$

$$\omega(t)=2\pi\left(f\pm\left(\frac{\Delta}{2}-\frac{\Delta t}{\tau_w}\right)\right) \tag{S5}$$

The $\omega(t)$ is the effective angular frequency function that can be inferred from Equation S3. B_0 is the main magnetic field. γ is the gyromagnetic ratio.

Equation S4 can be simplified when the frequency offset is negligible compared to the overall sweep range, so the B_1 amplitude profile $A(t)$ is irrelevant, and when the frequency sweep is linear.[9]

$$Q=\frac{(\gamma B_1)^2}{\left(\frac{d\omega}{dt}\right)}=\frac{(\gamma B_1)^2}{\left(\frac{2\pi\Delta}{\tau_w}\right)} \tag{S6}$$

However, this simplification cannot be applied under the circumstances of the low-field TOCSY experiment used here, as the sweep range of 10 kHz is not much greater than the γB_1 of 640 Hz. A low WURST pulse order provides a more gradual change in B_{eff} and increases adiabaticity, and it is viewed as a viable strategy to increase adiabaticity at B_1 stringency, such as in the experiments here.

Instead, the time dependent adiabaticity of the WURST pulses were calculated directly from equation S4.

The Q of the WURST pulse is calculated as a time-dependent function for the full sweep. The calculation was performed using a Python program. The minimum value at any time point is reported in the main text as the adiabaticity of the pulse, because the spin-lock at this point was the weakest. The ^{19}F spin receives slightly slower B_{eff} rotation because of its smaller gyromagnetic ratio compared to ^{1}H, so its adiabaticity is reported. Since the experiment was conducted with significantly less available B_1 amplitude when compared to conventional practice,[10] a sweep range of 10 kHz was adopted to give best results.

There are three straightforward ways from Equation S6 to achieve high adiabaticity, which are to increase B_1 or adiabatic pulse duration, or to decrease the sweep range. However, the experiments already implements the maximum B_1. In addition, the sweep range cannot be too narrow or the adiabaticity offset reduction is pronounced.[9] As a result, the strategies of using adiabatic pulse order

and duration to increase performance are shown in the discussion. The pulse amplitude is also intentionally decreased to demonstrate the effect of low adiabaticity.

4. Test Experiments

The resonance frequency miscalibration effect of the tailored pulses and the WURST pulse was tested by intentionally introducing a -100 Hz offset to the frequencies (Figure S3). The adiabatic pulse was used with an order of 2, duration of 200 ms and amplitude of 640 Hz, which were the same as the sufficiently powered reference experiments. Quadrature artifacts which could manifest as mirrored peaks were are not observed. Additionally, a spectrum with a DIPSI-2 isotropic mixing sequence with a duration of 36.84 ms and amplitude of 640 Hz was measured and referenced to the WURST pulse sequence (Figure S4).[5]

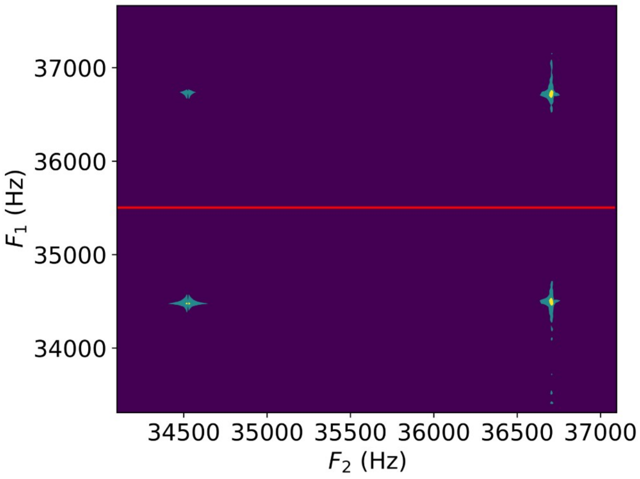

Figure S3: Test spectrum for resonance frequency miscalibration in which the WURST pulse used 200 ms duration, 640 Hz amplitude and N = *2. The WURST frequency center is marked by the horizontal red line.*

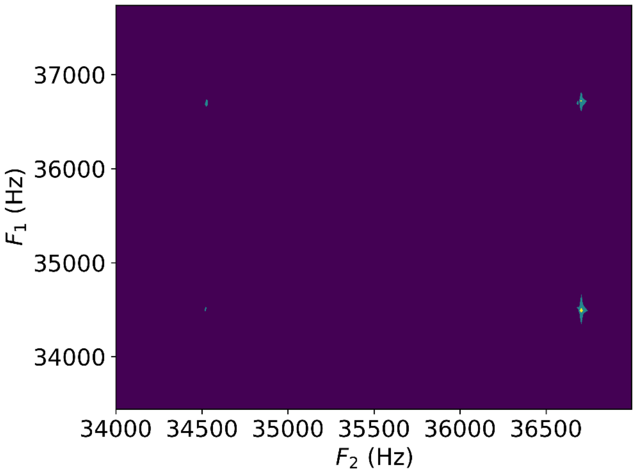

Figure S4: DIPSI-2 TOCSY spectrum in which the DIPSI-2 pulse is set with a γB_1 *amplitude 640 Hz and an overall duration of 36.84 ms.*

5. Isotropic Mixing Efficiency Measurements

Table S3: Isotropic mixing efficiency, calculated as cross peak intensity divided by the sum of cross and diagonal peak intensities, Eff = (P_{cross} / (P_{cross} + $P_{diagonal}$)) where P *is the instrumental SNR of the cross or diagonal peaks of the same nuclei on the direct axis. The calculation is performed separately for ^{1}H and ^{19}F peaks.*

Efficiency	WURST							DIPSI-2	
	200 ms, 640 Hz, N=2	500 ms, 640 Hz, N=2	800 ms, 640 Hz, N=2	200 ms, 640 Hz, N=6	200 ms, 640 Hz, N=16	200 ms, 240 Hz, N=2	200 ms, 120 Hz, N=2	36.84 ms	36.84 ms, miscal
Exp 1, ^{1}H	0.5013	0.5036	0.4995	0.4990	0.4973	0.4912	0.4606	0.6350	0.2617
Exp 1, ^{19}F	0.3031	0.2879	0.3149	0.3018	0.3041	0.2995	0.4839	0.6155	0.2342
Exp 2, ^{1}H	0.5018	0.5032	0.4988	0.4993	0.5195	0.4958	0.4974	0.6334	0.2669
Exp 2, ^{19}F	0.3060	0.2738	0.3312	0.3165	0.3057	0.3021	0.3910	0.5984	0.2831

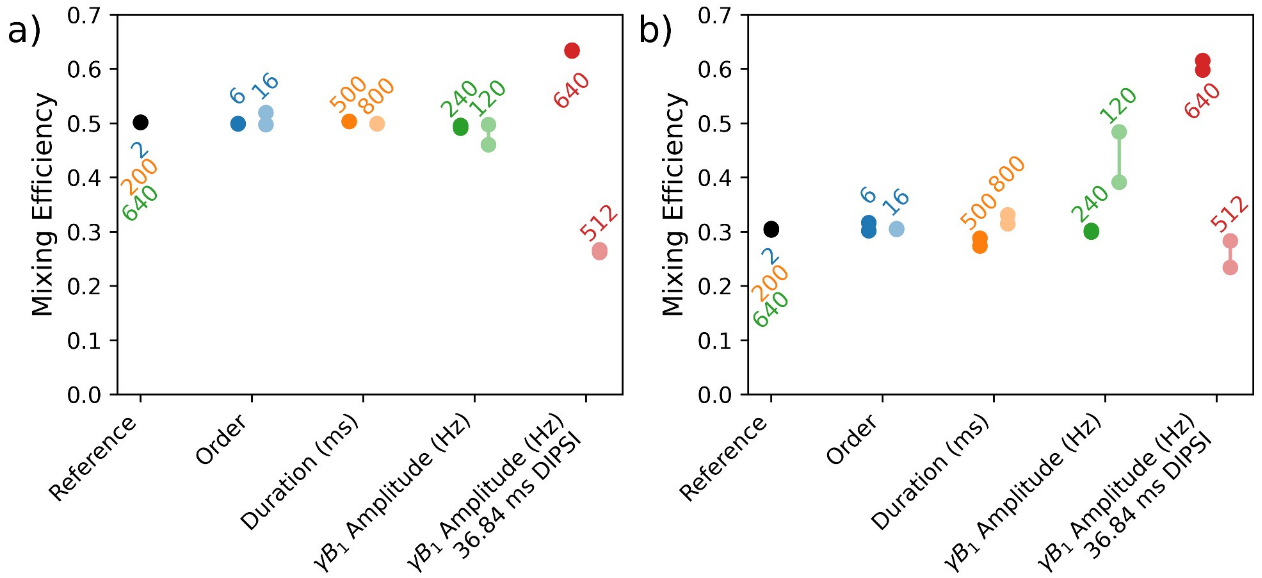

Figure S5: The receiving mixing efficiencies of the experiments different WURST and DIPSI-2 pulse setting of a) ^{1}H and b) ^{19}F peaks. The 200 ms duration, 640 Hz amplitude and N = 2 *WURST pulse is used as reference, colored black. Three control groups of pulse order, duration in milliseconds and* γB_1 *amplitude in Hz are compared to the reference with the variable parameter labeled and colored. Optimal and* B_1 *miscalibrated DIPSI-2 are compared to the WURST experiments, with* γB_1 *amplitude labeled. The two connected data are two repeats, listed in Table S3.*

6. Isotropic Mixing Efficiency Simulation

The time-dependent isotropic mixing efficiency was simulated in a Python program using the density matrix of a homonuclear two spin system in rotating frame, assuming J = 10 Hz. The simulation performed a numerical solution of the Liouville-von Neumann equation. The starting spin state was 100 % I_{1z}, and the I_{1z} and I_{2z} were tracked. The DIPSI-2 mixing sequence was tracked after every pulse. The WURST-2 was simulated by slicing the whole sweep in N slices, where in

every slice the amplitude and frequency of the adiabatic pulse was assumed to have negligible change. Therefore, the overall Hamiltonian of B_0 and B_1 were constants in each slice, and the same coherences were tracked at the end of every slice.

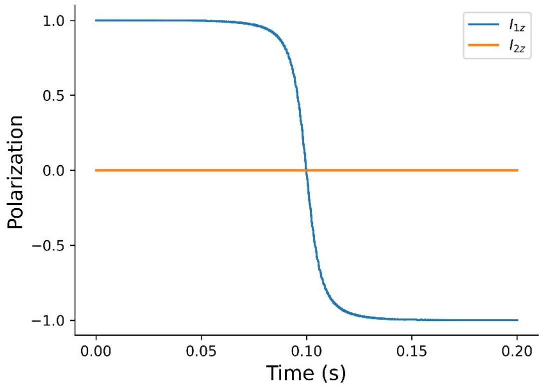

Figure S6: Simulated WURST-2 isotropic mixing on a two-spin system where J *= 0 Hz, showing an inversion profile.*

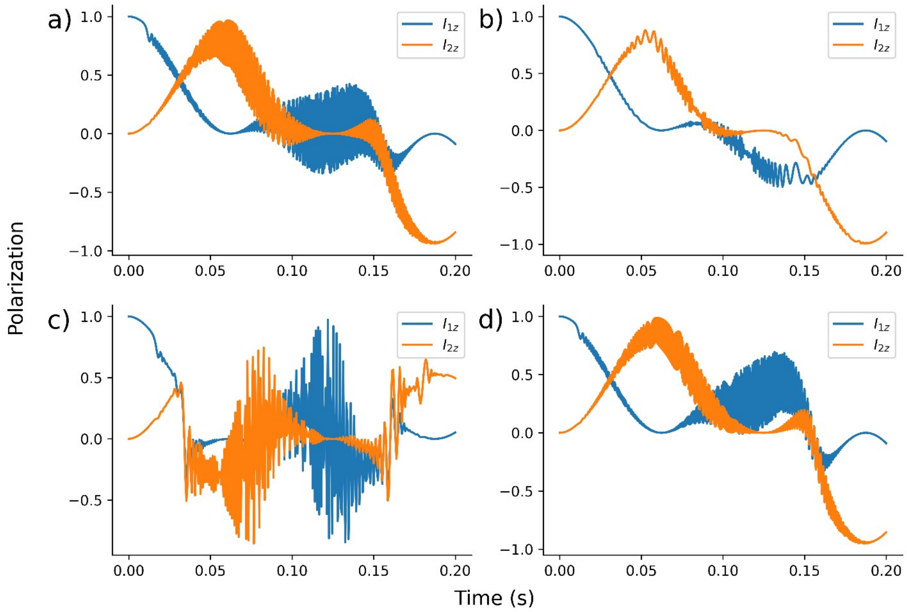

Figure S7: Simulated WURST-2 isotropic mixing of a two-spin system with J *= 10 Hz, using a number of time slices* N *of a) 1000, b) 400 and c) 300. The amplitude was 640 Hz, duration was 200 ms and order was 2. d) Simulated WURST-2 with the same parameters as a) but the amplitude set to 320 Hz, 50% of the level used in the experiments.*

The WURST-2 pulse swept over 10 kHz in 200 ms. The amplitude of the DIPSI-2 pulses and maximum amplitude of WURST-2 were equivalent to a B_1 field of 640 Hz. The simulation with no *J*-coupling (Figure S6) yielded the expected full passage inversion of I_{1z}. The simulation results when N = 1000 (Figure S7a) and N = 400 (Figure S7b) for WURST-2 were found to have insignificant differences. On the other hand, 300 slices (Figure S7c) were not sufficient. As a result, N = 400 was used in the offset profile simulation. The amplitude insensitivity of the WURST pulse is demonstrated by using 50 % pulse amplitude (Figure S7d) while still giving similar mixing curves.

The offset profile of isotropic mixing composes of the terminal status of I_{2z} of the time-dependent curve at 200 ms. It is plotted with respect to the offset of both I_1 and I_2 frequencies and shown in the main text in Figure 5.

7. Wide Range Isotropic Mixing Efficiency Simulation

The 2D isotropic mixing efficiency offset profiles of DIPSI-2 and WURST pulses were simulated and plotted to illustrate the performance of an adiabatic mixing pulse at a wider frequency range, for example, mixing between ^{1}H and ^{13}C, at varying field strength. N = 2 was used as the order of the WURST pulse, and the duration of the WURST pulse was 200 ms. The adiabatic sweep covered 0 to 1.1 times ^{1}H Larmor frequency. The efficiencies at offsets corresponding to ^{1}H-^{31}P, ^{1}H-^{13}C and ^{1}H-^{15}N are reported. The simulations included the experimental field strength of 0.86 mT, Earth field strength at 63 μT and 1% of experiment field strength, 8.6 μT. First, the *J*-coupling constant was kept at the same value of 10 Hz as above.

At 0.86 mT, corresponding to the field strength of the experiment, a WURST pulse with expanded sweep range for ^{1}H-^{13}C mixing would possess an adiabaticity of 1.28, too low to be adiabatic. Instead, a WURST pulse at 4.5 times the experimental B_1 was simulated (Figure S8). This pulse exhibits an adiabaticity of 16.59. The simulation showed an efficiency of 0.27 for ^{1}H-^{31}P, 0.058 for ^{1}H-^{13}C and 0.0093 for ^{1}H-^{15}N mixing. This amplitude was close to the minimum required. At 20% decreased amplitude, *i.e.* 3.6 times the experimental B_1, the adiabaticity drops to 8.00. At the reduced amplitude, the efficiencies were 0.21, 0.034 and 0.0073, respectively. In contrast, a DIPSI-2 pulse of 4.5 times the experimental B_1 did not achieve any significant ^{1}H-X mixing.

At 63 μT, the College Station, TX, Earth field strength, the WURST pulse at the experimental B_1 possessed an adiabaticity of 17.4 and showed 0.62 efficiency for ^{1}H-^{31}P mixing, 0.43 for ^{1}H-^{13}C and 0.13 for ^{1}H-^{15}N (Figure S9). This amplitude was also found close to the minimum requires, since at 20% decreased amplitude, the adiabaticity was 11.1 and the efficiencies were 0.44, 0.27 and 0.067, respectively. Likewise, the DIPSI-2 pulse of experimental B_1 did not achieve any significant ^{1}H-X mixing.

At 8.6 μT, corresponding to 1% of the experimental field strength, the WURST pulse at experimental B_1 possessed an adiabaticity of 1280 and showed 0.67 efficiency for ^{1}H-^{31}P, 0.52 for ^{1}H-^{13}C and 0.35 for ^{1}H-^{15}N mixing (Figure S10). This pulse amplitude was sufficient, since at 20% decreased amplitude, the WURST adiabaticity remained high at 819 and efficiencies were still 0.66, 0.51 and 0.34 respectively. The DIPSI-2 pulse of experimental B_1 achieved weak mixing of 0.028 for ^{1}H-^{31}P, 0.016 for ^{1}H-^{13}C and 0.016 for ^{1}H-^{15}N, about 3-5% that of WURST.

The influence of the ^{1}H-X coupling constant was investigated by performing an additional simulation with the coupling constant set to J = 140 Hz (Figure S11). It yielded a similar mixing efficiency profile compared to the J=10 Hz simulation (Figure S8). With identical adiabaticity, the efficiency was 0.26 for ^{1}H-^{31}P mixing, 0.058 for ^{1}H-^{13}C and 0.0088 for ^{1}H-^{15}N. This comparison suggests that the WURST mixing efficiency is insensitive to the coupling constants with sufficient mixing time.

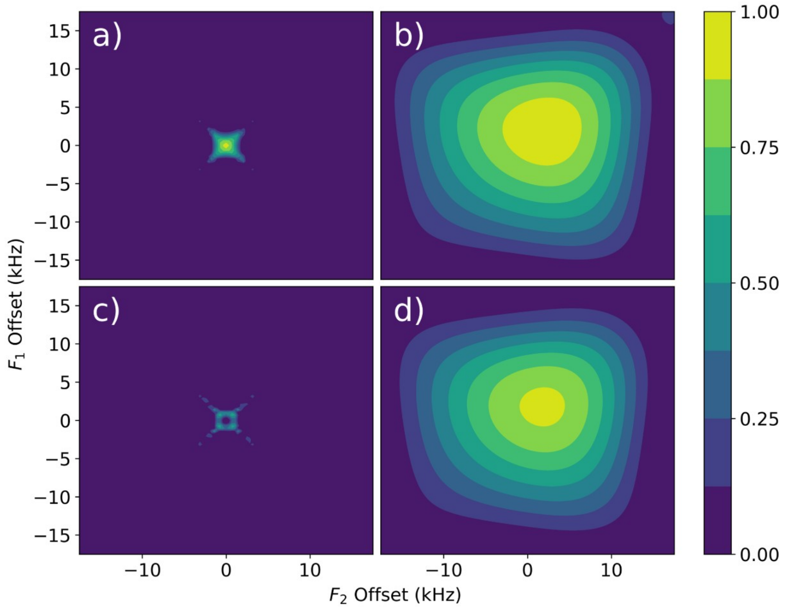

Figure S8: Simulated offset-dependent isotropic mixing efficiency profile at 0.86 mT of a) DIPSI-2, b) WURST pulse, c) -20% B_1 *miscalibrated DIPSI-2 and d) 20%* B_1 *WURST pulse. The pulses were simulated on a two-spin system where* J = *10 Hz. The reference DIPSI-2 pulse used a* γB_1 *of 2880 Hz, and the reference WURST used a pulse duration of 200 ms, order of 2 and a maximum* γB_1 *of 2880 Hz.*

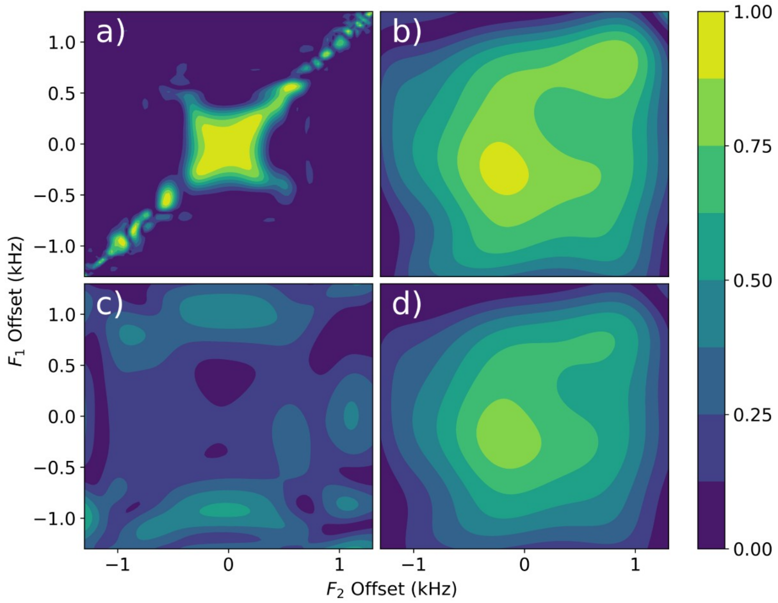

Figure S9: Simulated offset-dependent isotropic mixing efficiency profile at 63 μT of a) DIPSI-2, b) WURST pulse, c) -20% B_1 *miscalibrated DIPSI-2 and d) 20%* B_1 *WURST pulse. The pulses were simulated on a two-spin system where J = 10 Hz. The reference DIPSI-2 pulse used a* γB_1 *of 640 Hz, and the reference WURST used a pulse duration of 200 ms, order of 2 and a maximum* γB_1 *of 640 Hz.*

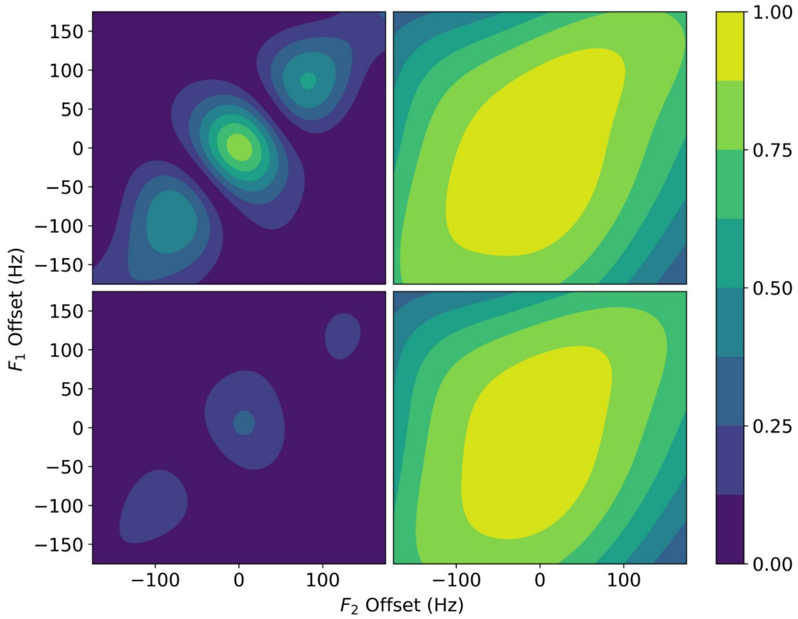

Figure S10: Simulated offset-dependent isotropic mixing efficiency profile at 8.6 μT of a) DIPSI-2, b) WURST pulse, c) -20% B_1 *miscalibrated DIPSI-2 and d) 20%* B_1 *WURST pulse. The pulses were simulated on a two-spin system where* J = *10 Hz. The reference DIPSI-2 pulse used a* γB_1 *of 640 Hz, and the reference WURST used a pulse duration of 200 ms, order of 2 and a maximum* γB_1 *of 640 Hz.*

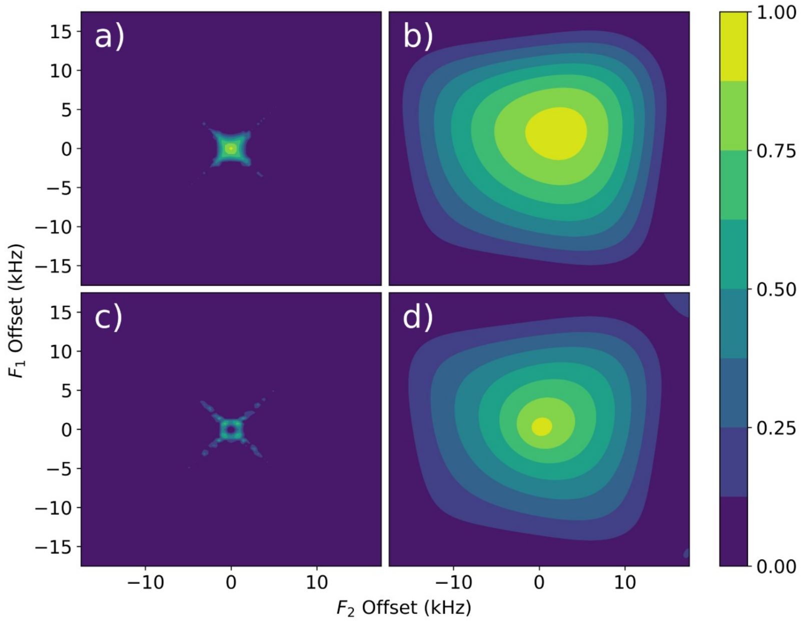

Figure S11: Simulated offset-dependent isotropic mixing efficiency profile at 0.86 mT of a) DIPSI-2, b) WURST pulse, c) -20% B_1 *miscalibrated DIPSI-2 and d) 20%* B_1 *WURST pulse. The pulses were simulated on a two-spin system where* $J = 140$ *Hz. The reference DIPSI-2 pulse used a* γB_1 *of 2880 Hz, and the reference WURST used a pulse duration of 200 ms, order of 2 and a maximum* γB_1 *of 2880 Hz. This single-bond* ^{1}*H-X coupling efficiency can be compared to multi-bond coupling where* $J = 10$ *Hz in Figure S8.*

8. References

(1) Zhu, Y.; Chen, C.-H.; Wilson, Z.; Savukov, I.; Hilty, C. Milli-Tesla NMR and Spectrophotometry of Liquids Hyperpolarized by Dissolution Dynamic Nuclear Polarization. *J. Magn. Reson.* **2016**, *270*, 71–76. https://doi.org/10.1016/j.jmr.2016.06.014.

(2) Zhu, Y.; Hilty, C.; Savukov, I. Dynamic Nuclear Polarization Enhanced Nuclear Spin Optical Rotation. *Angew. Chem.* **2021**, *60* (16), 8823–8826. https://doi.org/10.1002/ange.202016412.

(3) Kupce, E.; Freeman, R. Adiabatic Pulses for Wideband Inversion and Broadband Decoupling. *J. Magn. Reson. A* **1995**, *115* (2), 273–276. https://doi.org/10.1006/jmra.1995.1179.

(4) Cavanagh, J.; Rance, M. Sensitivity Improvement in Isotropic Mixing (TOCSY) Experiments. *J. Magn. Reson. 1969* **1990**, *88* (1), 72–85. https://doi.org/10.1016/0022-2364(90)90109-M.

(5) Rucker, S. P.; and Shaka, A. J. Broadband Homonuclear Cross Polarization in 2D N.M.R. Using DIPSI-2. *Mol. Phys.* **1989**, *68* (2), 509–517. https://doi.org/10.1080/00268978900102331.

(6) Zhang, Z.; Gautam, A.; Lim, S.-M.; Hilty, C. Analysis of Large Data Sets in a Physical Chemistry Laboratory NMR Experiment Using Python. *J. Chem. Educ.* **2023**, *100* (10), 4109–4113. https://doi.org/10.1021/acs.jchemed.3c00586.

(7) Kupče, Ē. Perspectives of Adiabatic Decoupling in Liquids. *J. Magn. Reson.* **2020**, *318*, 106799. https://doi.org/10.1016/j.jmr.2020.106799.

(8) Carvalho, J. P.; Pell, A. J. Frequency-Swept Adiabatic Pulses for Broadband Solid-State MAS NMR. *J. Magn. Reson.* **2021**, *324*, 106911. https://doi.org/10.1016/j.jmr.2020.106911.

(9) Tannús, A.; Garwood, M. Adiabatic Pulses. *NMR Biomed.* **1997**, *10* (8), 423–434. https://doi.org/10.1002/(SICI)1099-1492(199712)10:8%253C423::AID-NBM488%253E3.0.CO;2-X.

(10) O'Dell, L. A. The WURST Kind of Pulses in Solid-State NMR. *Solid State Nucl. Magn. Reson.* **2013**, *55–56*, 28–41. https://doi.org/10.1016/j.ssnmr.2013.10.003.
